# Real-Time fMRI Pattern Decoding and Neurofeedback Using FRIEND: An FSL-Integrated BCI Toolbox

**DOI:** 10.1371/journal.pone.0081658

**Published:** 2013-12-02

**Authors:** João R. Sato, Rodrigo Basilio, Fernando F. Paiva, Griselda J. Garrido, Ivanei E. Bramati, Patricia Bado, Fernanda Tovar-Moll, Roland Zahn, Jorge Moll

**Affiliations:** 1 Cognitive and Behavioral Neuroscience Unit and Neuroinformatics Workgroup, D’Or Institute for Research and Education (IDOR), Rio de Janeiro, Brazil; 2 Center of Mathematics, Computation and Cognition, Universidade Federal do ABC, Santo André, Brazil; 3 Institute of Physics of São Carlos, University of São Paulo, São Carlos, Brazil; 4 Institute for Biomedical Sciences, Federal University of Rio de Janeiro (UFRJ), Rio de Janeiro, Brazil; 5 Department of Psychological Medicine, Institute of Psychiatry, King's College, London, United Kingdom; University of Minnesota, United States of America

## Abstract

The demonstration that humans can learn to modulate their own brain activity based on feedback of neurophysiological signals opened up exciting opportunities for fundamental and applied neuroscience. Although EEG-based neurofeedback has been long employed both in experimental and clinical investigation, functional MRI (fMRI)-based neurofeedback emerged as a promising method, given its superior spatial resolution and ability to gauge deep cortical and subcortical brain regions. In combination with improved computational approaches, such as pattern recognition analysis (e.g., Support Vector Machines, SVM), fMRI neurofeedback and brain decoding represent key innovations in the field of neuromodulation and functional plasticity. Expansion in this field and its applications critically depend on the existence of freely available, integrated and user-friendly tools for the neuroimaging research community. Here, we introduce FRIEND, a graphic-oriented user-friendly interface package for fMRI neurofeedback and real-time multivoxel pattern decoding. The package integrates routines for image preprocessing in real-time, ROI-based feedback (single-ROI BOLD level and functional connectivity) and brain decoding-based feedback using SVM. FRIEND delivers an intuitive graphic interface with flexible processing pipelines involving optimized procedures embedding widely validated packages, such as FSL and libSVM. In addition, a user-defined visual neurofeedback module allows users to easily design and run fMRI neurofeedback experiments using ROI-based or multivariate classification approaches. FRIEND is open-source and free for non-commercial use. Processing tutorials and extensive documentation are available.

## Introduction

Humans can learn to modulate their own physiological responses, including viscero-endocrine and neural activity [1] especially when provided with contingent feedback signals in the form of brain-computer interfaces (BCI), a process that has been dubbed “neurofeedback” [2,3]. EEG-based neurofeedback has been extensively used in experimental and clinical investigation, including in epilepsy, attention deficit hyperactivity and affective disorders [2,4–6]. BCIs may allow paralyzed patients to control artificial limbs and speech devices ([7,8]; see [3] for an overview). In addition, BCIs based on invasive recordings hold promise for treating severe neurological deficits [9].

Critical advances in functional magnetic resonance imaging (fMRI) instrumentation and data processing [10] have paved the way for the implementation of real-time fMRI data analysis and neurofeedback [11–15]. Functional MRI-based neurofeedback (fMRI-NFB) enabled non-invasive modulation of brain regions at an unprecedented spatial accuracy, including deep brain structures that are virtually inaccessible by non-invasive methods. 

Combined with multivoxel pattern analysis or “brain decoding” techniques [16–18], fMRI-NFB holds great promise for experimental and clinical neuroscience. Real-time fMRI neurofeedback and multivoxel pattern analysis are especially promising to investigate brain states relying on the distributed networks underlying normal social cognition and emotion and their putative changes in psychiatric conditions [19-23]. Whereas multivoxel pattern analysis and neurofeedback can be combined to enable real-time fMRI-NFB brain decoding [12,19–21], the shortfall of freely available, standalone tools remains a major challenge for the widespread use of fMRI-NFB. Notable exceptions are AFNI [10], which includes the SVM-based 3dsvm plugin [19], as well as univariate real-time fMRI capabilities [10], TurboFIRE (http://hsc.unm.edu/som/neuro/lab/people.shtml), which allows real-time fMRI analysis based on GLM and sliding window correlations, and Turbo-BrainVoyager (Brain Innovation, The Netherlands)‎, a commercial package that provides real-time fMRI GLM analysis and neurofeedback functionalities (www.brainvoyager.com/TurboBrainVoyager.html). 

Here we introduce a new toolbox, FRIEND (Functional Real-time Interactive Endogenous Neuromodulation and Decoding), which integrates key functionalities, including (1) real-time fMRI preprocessing, (2) multivoxel pattern analysis and decoding by support vector machines (SVM) and (3) a flexible, user-defined neurofeedback module based on univariate (single ROI BOLD signal or dual ROI correlation), or multivariate SVM analyses, packaged within a standalone, user-friendly solution. This toolbox not only streamlines several processing steps, e.g. image registration, motion correction, spatial smoothing, GLM calculation, anatomically or functionally-defined ROI selection, but introduces optimized approaches for automatic map generation, calculation of dynamic signal correlation among ROIs using sliding windows, and for SVM training/classification. FRIEND is freely available for download at the Oxford FMRIB website (http://fsl.fmrib.ox.ac.uk/fsl/fslwiki/OtherSoftware) or directly from its repository (http://idor.org/neuroinformatics/friend) under an open-source license for non-commercial use. The toolbox was developed to address most technical and conceptual challenges in fMRI real-time classification of brain states (brain decoding) and neurofeedback. FRIEND does not require programming or scripting skills, and should be accessible to most students and researchers conducting fMRI research. In this article we describe the conceptual development and technical implementation of FRIEND, and illustrate some of its potential applications using real-time fMRI neurofeedback datasets of motor imagery and emotional elicitation tasks.

## Materials and Methods

### Ethics statement

All participants provided written consent for participating in these studies. This study and all data herein presented was approved by the local ethics committees (Copa D’Or CEP#137/09 and UFRJ CEP#159.709).

### FRIEND Toolbox Overview

FRIEND was developed at the Cognitive and Behavioral Neuroscience Unit, D’Or Institute for Research and Education (http://idor.org/neuroinformatics/friend), Rio de Janeiro, Brazil. The package was coded in Object PASCAL (Delphi^®^ 2007 and Lazarus 1.0.10) and C^2+^ (Microsoft Visual Studio® 2008 Professional and GNU Compiler Collection 4.8.1). FRIEND is multiplatform, running on Microsoft Windows^®^ (XP or later), Apple Macintosh (OS X 10.8 and above) and Linux (Debian, CentOS 6.4). A mid/high end workstation is required (e.g. PC: Quad-core i7, 8 GB RAM or above, Macintosh: Quad-core Intel Core i5, 8 GB RAM or above) in order to enable smooth online data preprocessing, classification and contingent stimulus delivery. FRIEND employs multithread coding for speeded up processing in multiple core workstations. This feature is implemented by calling embedded FSL routines (http://www.fmrib.ox.ac.uk/fsl/) into different threads. The original FSL codes were not modified for parallel processing. All steps of image registration, motion correction, feature selection (based on either SVM or general linear model [GLM] “functional localizers”, or on *a priori* ROIs) and SVM classification can generally be performed within a TR of 1.5 seconds or less (single-shot EPI, 64x64 to 80x80 matrix, 22-37 slices.

**Figure 1:**
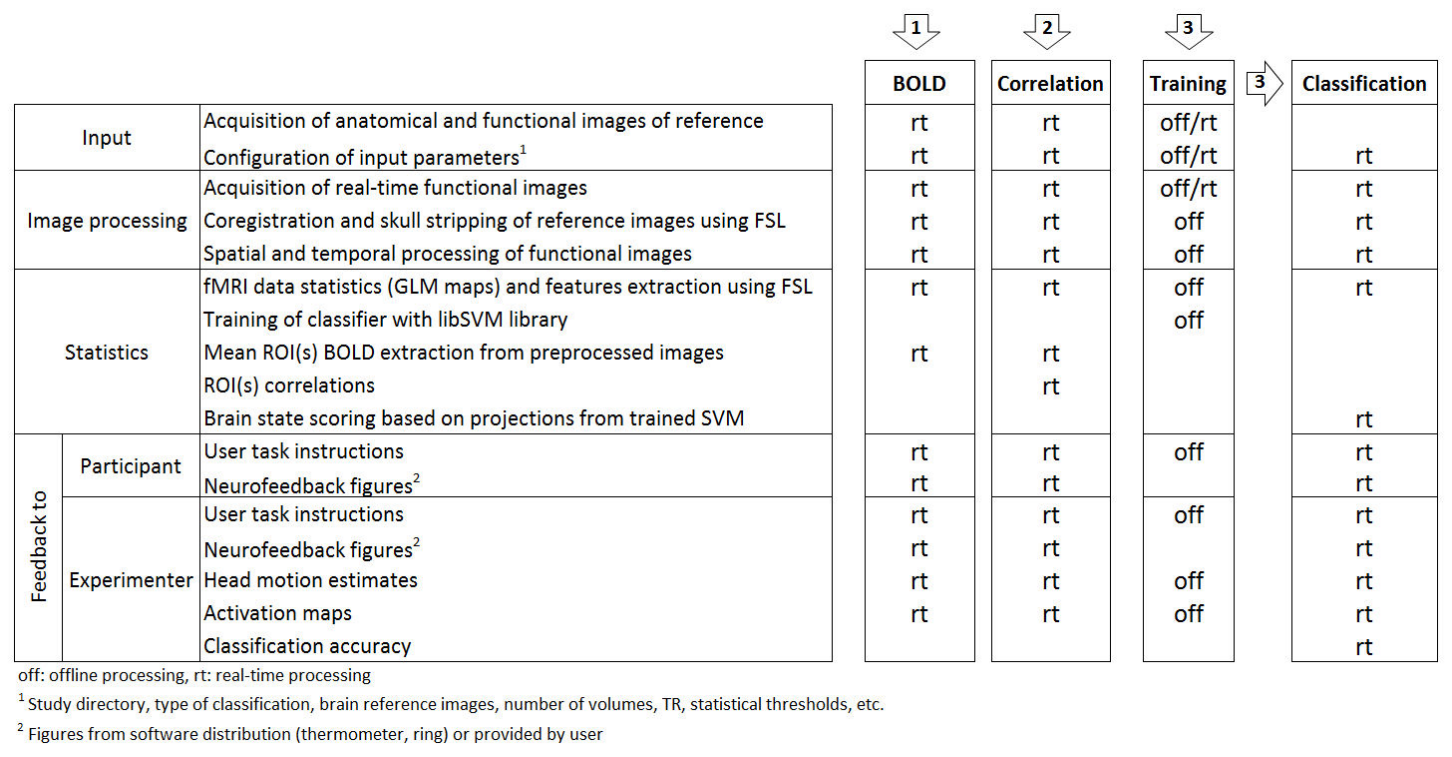
Flowchart of three FRIEND processing pipelines for neurofeedback. (1) BOLD level real-time display from pre-defined ROIs; (2) Real-time functional connectivity neurofeedback based on the correlation between the signals from different ROIs; (3) Support Vector Machine based neurofeedback, defined on the basis of projected values onto the discriminative hyperplane.

The real-time preprocessing module includes options for univariate (ROI-based) and multivariate SVM data analysis [24,25] and/or classification, coupled with the visual neurofeedback module. This enables participants to use their own local (single ROI or combined ROIs) or distributed brain signals (correlation among ROIs or multivoxel pattern-based brain decoding using SVM) to modulate performance in a wide range of behavioral (e.g., motor task), cognitive (e.g., motor imagery) or emotional tasks (e.g., basic, social or moral emotions). Figure 1 shows a flowchart describing FRIEND’s main pipeline elements.

The FRIEND toolbox currently embeds components from the FSL ([26]; http://www.fmrib.ox.ac.uk/fsl/) and from the libSVM ([27]; http://www.csie.ntu.edu.tw/~cjlin/libsvm/) libraries, both freely available packages with stable releases that have been extensively validated by the scientific community. FRIEND also incorporates a number of modules and routines designed specifically to simplify the conduction of real-time fMRI neurofeedback experiments, while allowing extensive control of parameters and quality control. Furthermore, to allow for controlled studies, FRIEND offers the option of running an experiment with contingent (“real”) or non-contingent (e.g., random or non-informative) neurofeedback. Thus, participants may be randomly assigned to a neurofeedback or to a control / non-feedback group.

### Data Acquisition and Processing Overview

**Figure 2:**
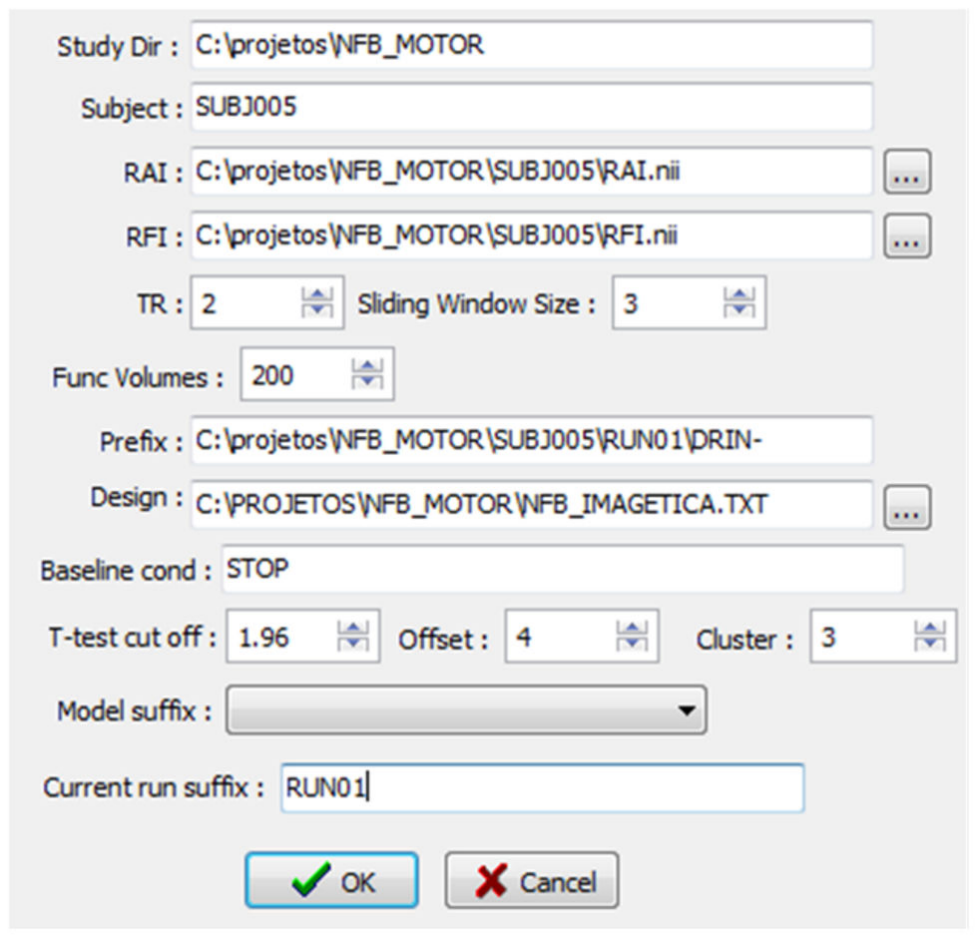
Typical parameters for a study session in FRIEND (anatomical and functional volumes of reference, number of volumes, and statistical thresholds, among others). Additional parameters (e.g., % of higher voxels for GLM feature selection, inclusion of motion parameter variables in the GLM model, FWHM values) can be modified by editing an input text file.

Data collection begins with the acquisition of a high-resolution gradient-echo T1-weighted structural anatomical volume (reference anatomical image, RAI) and one high signal-to-noise echo-planar (EPI) volume (reference functional image, RFI), which are used as image registration references. Functional images are then obtained using the real-time acquisition pipeline. The experimental design is described in an ASCII design file while other parameters (preprocessing parameters, type of feature selection, if any, and feedback characteristics) are entered into the software interface window (Figure 2). 

FRIEND's real-time functionalities inherently require proper access to the functional volumes as soon as they are acquired. Thus, real-time fMRI data (single EPI volumes) must be available from the MR scanner in a suitable data format immediately following reconstruction. It should be noted that FRIEND does not access the imaging data directly from the scanner. Instead, it reads the data from a shared folder where the reconstructed images are saved in real time. To the best of our knowledge, real-time data reconstruction and export (or online access to reconstructed images) is currently available from at least three of the main manufacturers (Philips Medical Systems, Siemens Medical Solutions and GE Medical Systems). Siemens has a built-in tool, which is standard starting from release VB15 [20]. Philips provides the DRIN-dumper as a clinical research tool, and real-time solutions for GE scanners are also available ([28]; see also https://github.com/cni/rtfmri). In addition to the proprietary software mentioned above, there are also other options for real-time data handling (e.g., FieldTrip, http://fieldtrip.fcdonders.nl). So far, FRIEND has been tested with Philips and Siemens scanners. 

In its current implementation, FRIEND requires at least one condition of no interest (i.e., baseline), which should be included between blocks of the main experimental conditions, in order to allow for online signal normalization and detrending. These steps are important to minimize the effects of MRI signal drifts (see 12). 

**Figure 3:**
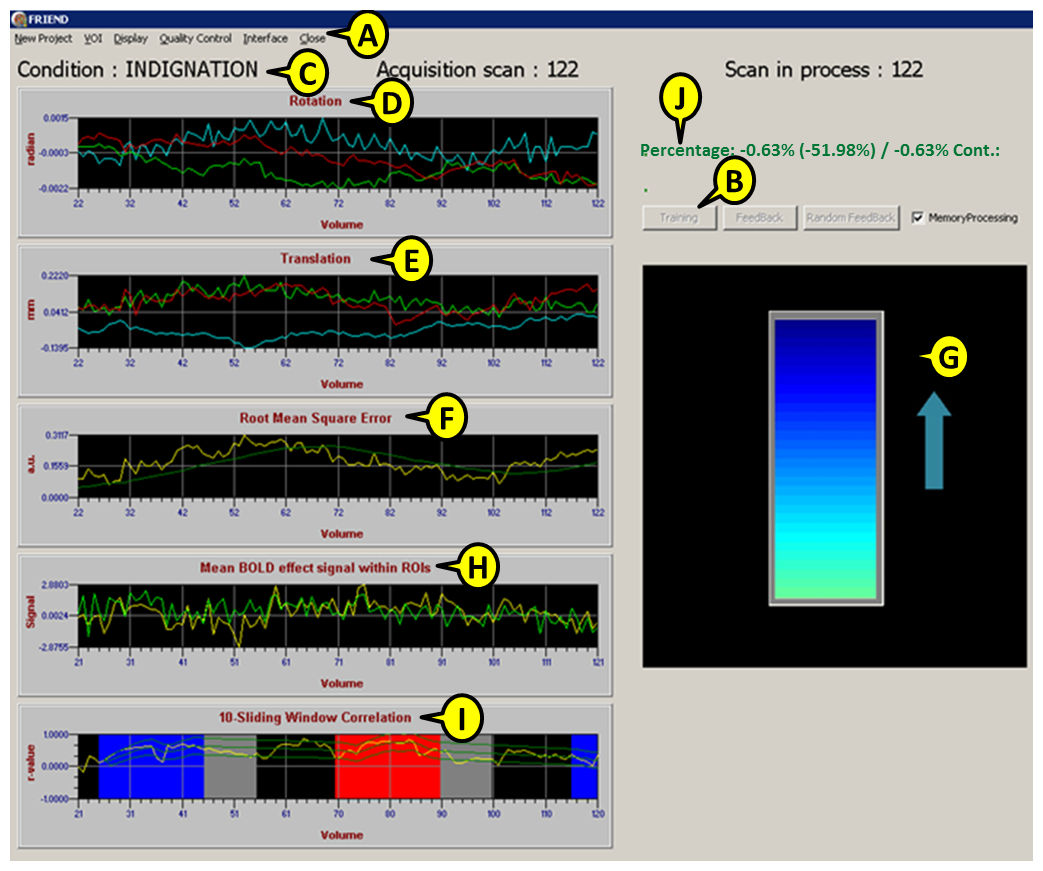
FRIEND’s control window, including: the main menu (A), training and feedback buttons (B), current experimental condition (C), rotation in radians (D), translation in mm (E) and root mean square error from motion parameters (F). User-defined neurofeedback stimuli to be presented to participants (a thermometer in this case) are displayed when the feedback option is selected (G). For single ROI processing, time-course, mean signal within specified ROIs, signal change and condition blocks will be shown (H). In the case of sliding-window ROI correlation analysis, a similar graph shows the level of correlation, sliding window size and upper and lower bounds of correlation targets (I). During the SVM classification sessions, the interface shows the classification phase, the current scan and the model-based cumulative classification accuracy (J).

The graphical user interface (GUI) control window includes online charts for functional image registration to the reference volume (including translation, rotation and root mean square error [RMS]). Accuracy estimates (correct classification of individual functional volumes when using SVM), normalized signal in selected ROIs (i.e., BOLD changes) and sliding window correlations among ROIs can be dynamically evaluated in the same control window during real-time fMRI (Figure 3).

### Real-time Image Preprocessing

The first step consists of an affine co-registration of RFI to RAI (12 degrees of freedom, using the FLIRT routine (http://fsl.fmrib.ox.ac.uk/fsl/fslwiki/FLIRT). This transformation is subsequently used to adjust incoming EPI images during the functional runs both to the RFI (for pipeline processing) and RAI (for real-time activation map overlay) via the real-time motion correction routine based on FSL routines. Motion estimation and correction can be performed using the embedded MCFLIRT ([29]; http://fsl.fmrib.ox.ac.uk/fsl/fslwiki/MCFLIRT) library (-cost is set to normcorr and interpolation to trilinear sampling, which are the default options in MCFLIRT). Following image registration, spatial Gaussian smoothing of the EPI volumes based on a user-defined FWHM parameter can also be carried out. In order to minimize MRI signal trends, voxel intensities are mean-corrected by the average signal from the previous baseline condition, specified in a design matrix file.

### Functional Localizers and Feature Selection

When using ROI or SVM-based neurofeedback, users may opt for running General Linear Model (GLM)-based statistics [30] on the initial dataset (e.g., first functional run) to be used as a functional localizer for single-region neurofeedback, for dual-region correlation analysis or before SVM training. This step employs embedded routines from the FSL library (*feat_model* and *fsl_glm, see* http://fsl.fmrib.ox.ac.uk/fsl/fslwiki/FEAT), allowing for *a priori*-defined statistical contrasts, which can be used for optional feature selection/masking of relevant voxels identified by a functional localizer or training session (see [31]). It is important to note that because this is a feature selection step, the GLM is carried out off-line (not in real-time) after the first run (“training session”). This is an important step for the following reasons: (i) for single ROI neurofeedback or dual-ROI real-time correlation, using a percentage of the more active voxels within selected anatomical ROIs can better capture individual differences; (ii) whole brain classification analysis leads to high dimensionality of the data, including confounders and irrelevant variables, so a feature selection step (e.g., using a combination of *a priori* ROIs, GLM and/or SVM-based thresholded maps) helps reducing dimensionality; and (iii) these procedures minimize the possibility that artifactual or uninformative voxels bias the results. 

### Support Vector Machines (SVM)

#### Training SVM Classifiers

The rationale for the use of SVM is its intrinsic ability to deal with the typical fMRI datasets, which contain typically tens of thousands voxels, i.e., when the number of features far exceeds the number of measurements. Ultimately, the goal of machine learning methods applied to fMRI data is to maximize the ability to make predictions about new, unobserved data, i.e., to allow generalization from observed data (“training”) to new datasets [32,33].

In FRIEND’s control window (Figure 3), when the “training” checkbox is selected, the SVM classifier will be initially trained with brain activation patterns associated with the specified conditions of interest in the training fMRI dataset. In addition, in order to increase the signal-to-noise ratio, each example is built by computing an average volume over three (or another user-defined number) previous volumes (sliding window average).

**Figure 4:**
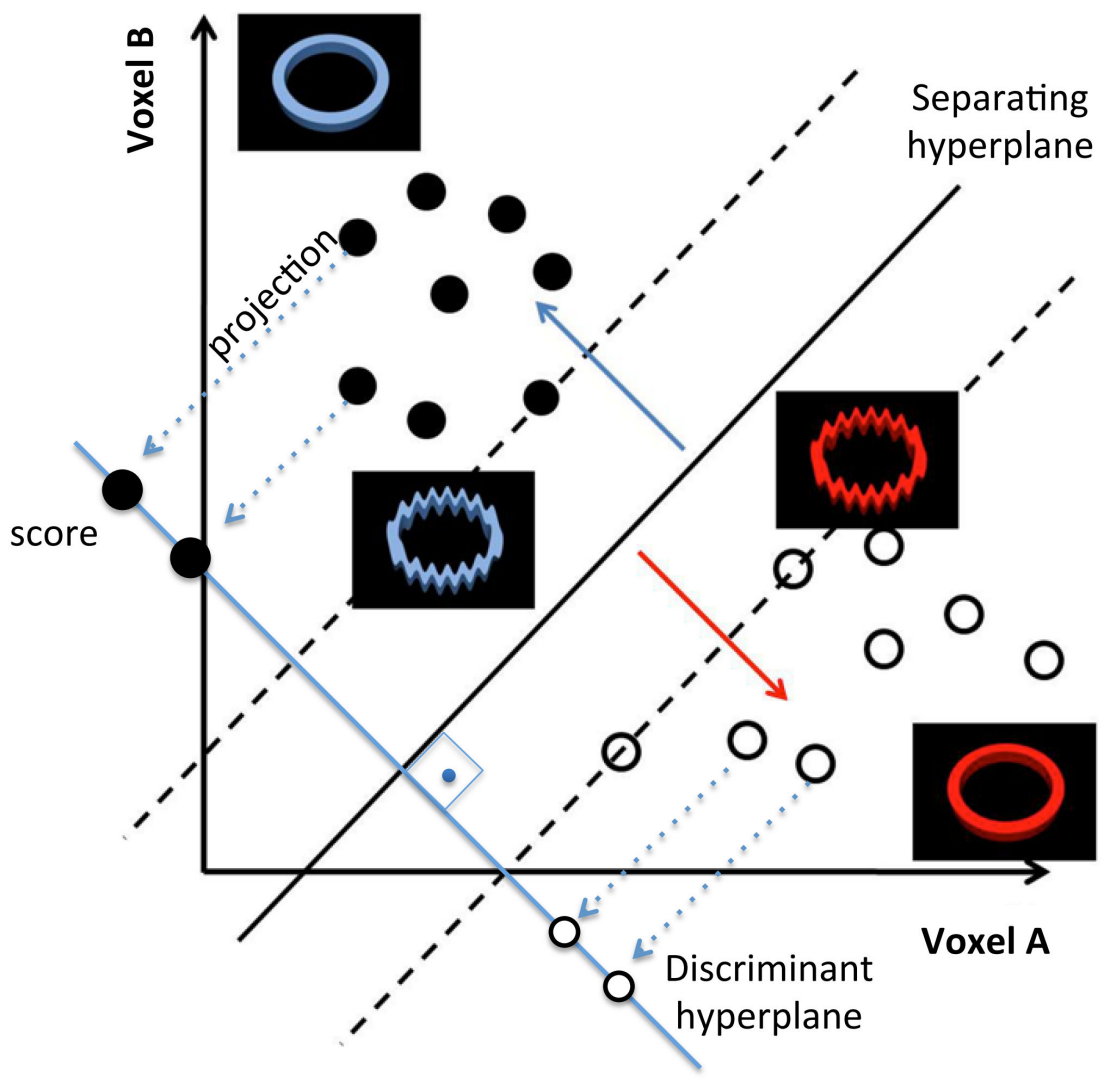
Illustration of how neurofeedback stimuli are defined based on the calculated projections on the SVM discriminant hyperplane. The black and white circles are observations of two different types of stimuli (e.g., positive and negative emotional condition). The basic concept is that after training a two-class linear SVM, a discriminant hyperplane is defined (in light blue). Next, each new fMRI volume is projected on this hyperplane (decision function) and a score is attributed, reflecting the relative distance from the classification boundary (intersection with separating hyperplane). This score is then categorized in order to determine which visual image will be displayed to the participant as a feedback.

The main concept behind the two-class SVM methodology is to determine a mapping from input data (activation pattern) to output experimental condition in order to correctly classify it. Once this function is estimated, it can be used to obtain scores for predictions of the classes of new observations [12], based on their input data (see Figure 4; [34,35]). The input data is the normalized BOLD signal intensity of input voxels.

The brain voxels of an fMRI image volume are first mapped onto an input vector *x*, and this vector is then labeled according to the respective experimental condition when this scan was acquired [19,20]. This initial data is used to train the classifier to discriminate between the experimental conditions of interest (currently, a two-class SVM classifier is implemented). The trained SVM is then used in the subsequent brain decoding sessions (testing sessions), in which participants engage in the same tasks and conditions of interest. 

#### Real-time Classification and Neurofeedback

After training a SVM on the initial dataset, predictions about the current cognitive/neural state of the subject can be made in real-time based on incoming fMRI image volumes. At this stage, neurofeedback is delivered by presenting visual feedback stimuli that are contingent on SVM classification. Although the classification is based on categorical output data, linear SVM can provide the distance of a new observation to the separating hyperplane, the classification boundary between conditions [35]; this projection (“decision value”) is then used to define the neurofeedback display. The projection of a new image volume on the discriminating hyperplane is given by (*x* ^*T*^ *w*+*b*), where *w* is a vector containing the hyperplane coefficients and *b* is a constant. In other words, the relative position of the input data projection to the classification boundary of the discriminative hyperplane is the measure that will define which figure (from a bitmap-grid stimulus set) will be displayed as a proxy of the underlying cognitive state of the participant. Further information about real-time classification/projection can be found in [12,19,34,35]. 

**Figure 5:**
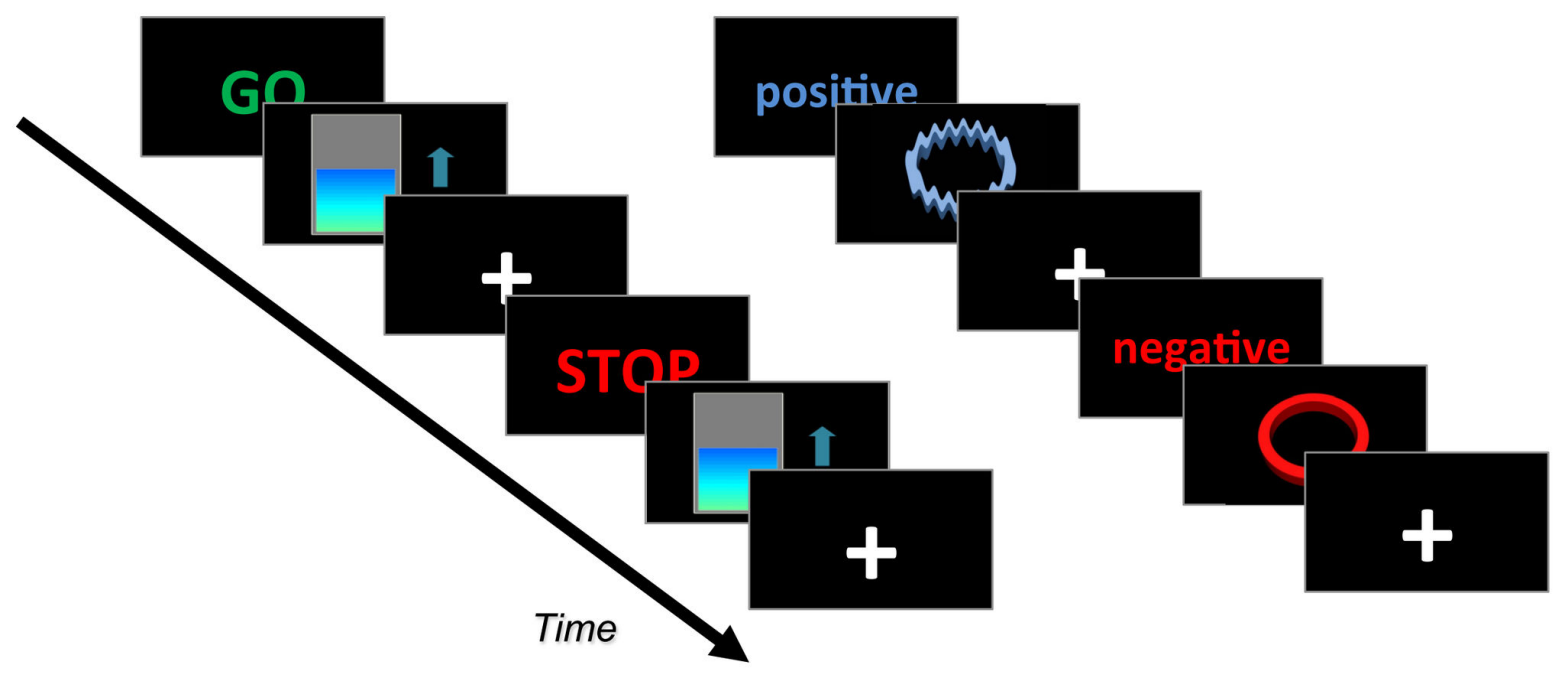
Example of feedback figures displayed in motor imagery (left) and emotional (right) neurofeedback protocols. FRIEND provides default neurofeedback figures (thermometer and rings), but user-defined ones may be used instead. The displayed words (GO/STOP and positive/negative) are cues for the specific task to be performed by participants.

**Figure 6:**
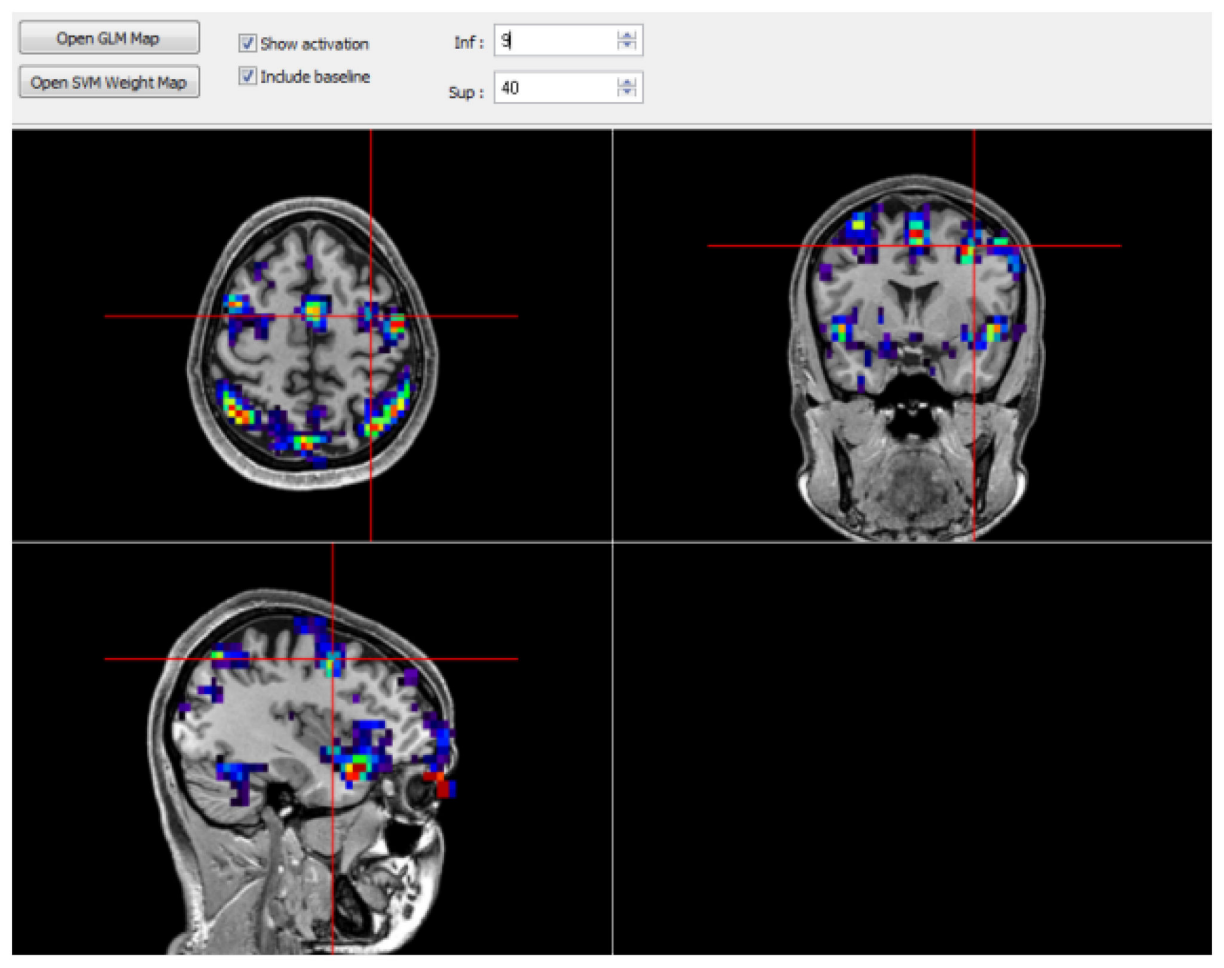
Real-time brain activation mapping, depicting the ratio [(average BOLD signal of the ROI during the three last scans) – (average BOLD signal of the ROI during the previous baseline condition)] / (average BOLD signal of the ROI during the previous baseline condition) for each voxel on the participant’s native space using an arbitrary image threshold.

In Figure 5 (right panel), the shape of the ring changes progressively from a distorted to a perfect ring according to the two-class SVM classification (decision function values). In this example, the most distorted shape is associated with incorrect classification, and the progressively smoother rings are associated with increasing distance of the correctly classified example from the SVM decision boundary. Increasing distance from the SVM decision boundary indicates that the activation pattern is more distinctive of one category (cognitive state) as compared with the other. Figure 6 depicts the display interface for real-time activation maps (image voxel intensity of current scan normalized by the previous *n*-averaged baseline condition images, which can be scrolled in real-time).

### ROI-based Neurofeedback

In the case of model-driven experiments, FRIEND allows the use of ROIs not only for real-time visualization of online brain activity but also for ROI-based neurofeedback. The GUI allows selecting ROIs from standard atlases (MNI, AAL, etc), from a mask file or from the GLM results of a functional localizer scan, which can be saved as ROIs for subsequent use. A moving-average BOLD signal from these regions can then be displayed (e.g., as a thermometer or a moving ring). As demonstrated in previous studies, participants can modulate BOLD activity of specific ROIs, guided by neurofeedback signals [11,28,36]. The basic concept is to use a block-design paradigm in which participants are instructed to try to increase or decrease BOLD signal averaged within an ROI, with the aid of a feedback display (see Video S1). The feedback values are given by the ratio [(average BOLD signal of the ROI) – (average BOLD signal of the ROI during the previous baseline condition)] / (average BOLD signal of the ROI during the previous baseline condition) rescaled to the interval 0-100%.

Figure 5 depicts two experimental designs using a thermometer and rings as feedback. Users may easily create and specify their own visual stimuli (JPEGs) to be employed as contingent feedback signals. In Figure 5 (left panel), the thermometer level is specified by the change in ROI-based image intensity of the current EPI image, normalized by the signal average of the *n*-preceding baseline volumes (*n* being the number of volumes to be averaged in the preceding block of the user-defined baseline condition).

### ROI-based Functional Connectivity Neurofeedback

FRIEND also allows functional connectivity-based neurofeedback using a sliding window and Pearson correlation coefficients of the signal between two ROIs. In the current version, only two ROIs are employed, thus whole brain functional connectivity maps are not available in real-time (though this feature can be implemented by advanced users). This approach enables experiments probing the effects of endogenous modulation of the connectivity between user-defined ROIs (including cortico-subcortical connectivity that cannot be assessed using non-invasive EEG-based methods). At each new volume acquisition, the coefficient is iteratively calculated over the last *L* scans (a user-defined parameter). To accomplish this, the mean intensity $r\bar{o}i=\sum_{i=1:m}x_{i}/m$ is calculated over the *m* voxels of the ROI, $r\bar{o}i$ at each time point *t* for subsequent calculation of the ROI mean $r\bar{o}i=\sum_{t=1:L}r\bar{o}i_{t}/L$. Thus, for ROIs A and B, the Pearson correlation coefficient over a *L*-sliding window at time *t* is:

$$\rho {\left(A,B\right)}_{t}=\frac{\sum_{k=1}^{L}\left({\bar{A}}_{t-k}-\bar{A})({\bar{B}}_{t-k}-\bar{B}\right)}{\sqrt{\sum_{k=1}^{L}{\left({\bar{A}}_{t-k}-\bar{A}\right)}^{2}}\sqrt{\sum_{k=1}^{L}{\left({\bar{B}}_{t-k}-\bar{B}\right)}^{2}}}$$

Our pilot studies indicate that more stable values of *ρ*(correlations) are obtained with *L=*[*10,…,15*]. This real-time functional connectivity measure can then be displayed as a feedback to the participant via user-defined visual cues (e.g., a thermometer). The mean value of the time-varying correlation scale (used to set the midline value of the feedback thermometer) employs a sigmoid-weighting discounting function (slope=1), which provides estimates that are more influenced by more recent values, relative to earlier ones (the number of volumes entered in this weighting function can be set by the user, but our experience suggests that a value of 10 volumes may be adequate). The upper and lower bounds of the correlation scale (which define the top and bottom levels of the thermometer) are defined on the basis of the calculated standard deviation of the correlation coefficients over the last L (e.g., 10) volumes. The multiplier of standard deviations is set to 1 by default, but can be changed as well. This provides a smooth and flexible control of the feedback thermometer feedback, and a more “natural” experience for participants whilst they attempt to modulate their own ROI-based correlations. An illustration of functional connectivity neurofeedback is shown in Video S2.

### Performance Optimization and Quality Monitoring

All image processing steps, including network communication and image transfer, image registration, feature selection and post-processing (based on single ROI BOLD, SVM or dual-ROI sliding correlation) and neurofeedback GUI display can be performed in under 1.5 seconds (generally within 1 second) on a proper workstation. For this purpose, a number of optimizations were conducted. 

In the Windows version, a DLL containing the FSL 4.1 commands was built to enable full control of command execution. Another reason to build a DLL is the simplified creation of functions related to the pipeline that receives internal memory data structures. This avoids excessive read/write files from disk by exchanging between functions instead of files, by using pointers to already allocated memory, leading to improved performance. Furthermore, having one DLL file replacing sets of different binaries is another advantage. The libSVM DLL was incremented with functions that enable direct reading of Analyze/NIfTI files and of memory data structures. Additionally, an experimental, optional automatic motion detection routine was implemented in FRIEND, based on root mean squared deviations (RMS) from a moving average over *n* scans (currently set to 40, according to our initial experience). This feature may be useful both to allow the experimenter to monitor a participant’s motion online and to notify the participant if he/she is moving beyond tolerable ranges during image acquisition. The threshold for the excessive movements is user-defined, but we are currently employing an RMS threshold of .4 (absolute deviation from the mean RMS), based on Jenkinson [37] and on our own piloting observations. Furthermore, this same threshold can be used by FRIEND to automatically discard volumes associated with head movement events, therefore minimizing contamination of single ROI, correlation or SVM estimates during real-time fMRI neurofeedback experiments. A similar approach of discarding unreliable scans has been employed in a recent study [38]. Furthermore, when significant motion is detected, FRIEND’s motion detection module communicates with the feedback module, “freezing” the feedback (i.e., the displayed ring or thermometer level), therefore visually informing participants about their own excessive movement (this is illustrated in Video S2).

In terms of performance, considering the acquisition of EPI volumes with 64x64x22 voxel resolution (3.75x3.75x5mm using an FOV=240mm) and whole brain analysis, and employing a PC Intel Core i7 3930v (12 cores), 16GB RAM, SSD 128GB, the processing time for each step was approximately as follows: head motion correction = 562ms; SVM training (including GLM for feature selection) = 10s; SVM testing < 100ms.

## Illustrative Applications

Below we provide illustrative examples of the three main types of real-time fMRI neurofeedback protocols currently available with FRIEND. These examples comprise the results of three typical participants for each type of fMRI-NFB protocol currently being conducted in our institution. We chose to report single participants for the sake of clarity and illustration. They are nonetheless fairly representative of results currently being obtained in group studies.

Participants were scanned on a 3T Achieva system (Philips Medical Systems, The Netherlands) equipped with gradients capable of 80mT/m amplitude and 200mT/m/ms slew rate, using an 8-channel head coil. A standard 2D gradient-echo EPI sequence was employed using the following parameters: TR/TE=2000/30ms, FOV=240x240mm^2^, matrix=64x64, slice thickness=5mm, 22 slices, EPI direction = AP, with SPIR fat saturation.

### Motor Imagery Data: ROI based neurofeedback

Three hundred EPI volumes per session (three sessions) were acquired in this experiment. Participants were instructed to perform a motor imagery task based on a finger tapping sequence using their right hand. The paradigm consisted of a block design in which blocks of motor imagery (15 volumes each) alternated with rest (also 15 volumes). Task instruction and feedback stimuli were displayed using an LCD monitor visualized using a mirror attached to the head coil. The neurofeedback protocol required the participant to increase the level of the thermometer while performing the imagery task, which evoked BOLD increases in the left lateral premotor cortex among other regions related to motor control. The ROI (left premotor area) was chosen based on a template from a meta-analysis [39]. Three-dimensional locations and boundaries of motor and premotor cortices were based on the same meta-analysis. During the functional localizer session, only the instruction cues were briefly presented to the subject (“STOP” for rest, “GO” for motor imagery). 

In this example, the participant (male, 31 y/o) was successful in increasing BOLD signal in his left premotor cortex, with the activation cluster size extension increasing progressively across neurofeedback sessions (number of active voxels: 24, 25 and 38, respectively). This type of neurofeedback approach is rather straightforward and can be useful in a number of studies, including clinical trials. The ability to use fMRI neurofeedback to increase activation in the premotor cortex has potential clinical applications, such as for motor rehabilitation in stroke patients [40]. An illustrative video is provided as a supplementary material (Video S1). 

In order to evaluate the real-time preprocessing effects on the ROI activation index (calculation described previously in subsection 2.6), we compared our real-time implementation to the offline processing using standard FSL-FEAT. This evaluation was based on the data from three single subjects. The correlations between real-time and offline preprocessing activation indexes (over time) were above 0.99 in all participants.

### Emotional memory data: SVM-based brain decoding

In this example, participants were asked to identify two types of emotionally salient autobiographical memories: positive and negative. These memories were used as the main conditions. As a baseline condition, neutral memories (e.g., daily routines) were employed. Participants were cued with visually presented keywords to engage in their positive, negative or neutral memories. In the functional localizer scan, participants only saw the cues of the above conditions and performed the task without feedback. A two-class SVM was trained to discriminate between the positive and negative emotional memories based on the distributed voxel patterns of BOLD signals from EPI preprocessed images. In the subsequent classification runs, the same design was employed, but this time combined with neurofeedback. The experiment comprised four runs (first, SVM training followed by three classification/neurofeedback sessions), with two hundred ninety-six volumes acquired in each run. A block-design was employed, and each emotional block (4 positive and 4 negative per run; 22 volumes each) was interleaved with the neutral condition (8 blocks per session, 15 volumes each) for disengagement from the preceding condition and for signal normalization/detrending purposes. The temporal sliding window for scans averaging was 3 volumes (6 seconds). Before training the SVM, a voxel selection procedure was carried out off-line by calculating a GLM map and then thresholding it for absolute t-values greater than 5.5 (pooling voxels passing this threshold for all pairwise contrasts among the neutral, positive and negative conditions). No anatomical, *a priori* masks were used in this experiment. The feedback values were based on the SVM decision values for each newly acquired dataset (average of the last 3 volumes). Note that only classification must be carried out in real time, since SVM training (and voxel selection using GLM) are performed offline on the training run. During the neurofeedback sessions, the task cue (keyword pointing to the specific positive, negative or neutral memory) was briefly displayed and then followed by the time-varying feedback stimuli (in this example, the ring figures). Depending on the value associated with the decision function, the ring became progressively more distorted or smoother. Real-time feedback was thus contingent on how well a participant’s current pattern of brain activity approached the target defined during the training session (positive or negative emotional memory; see Video S3).

**Figure 7:**
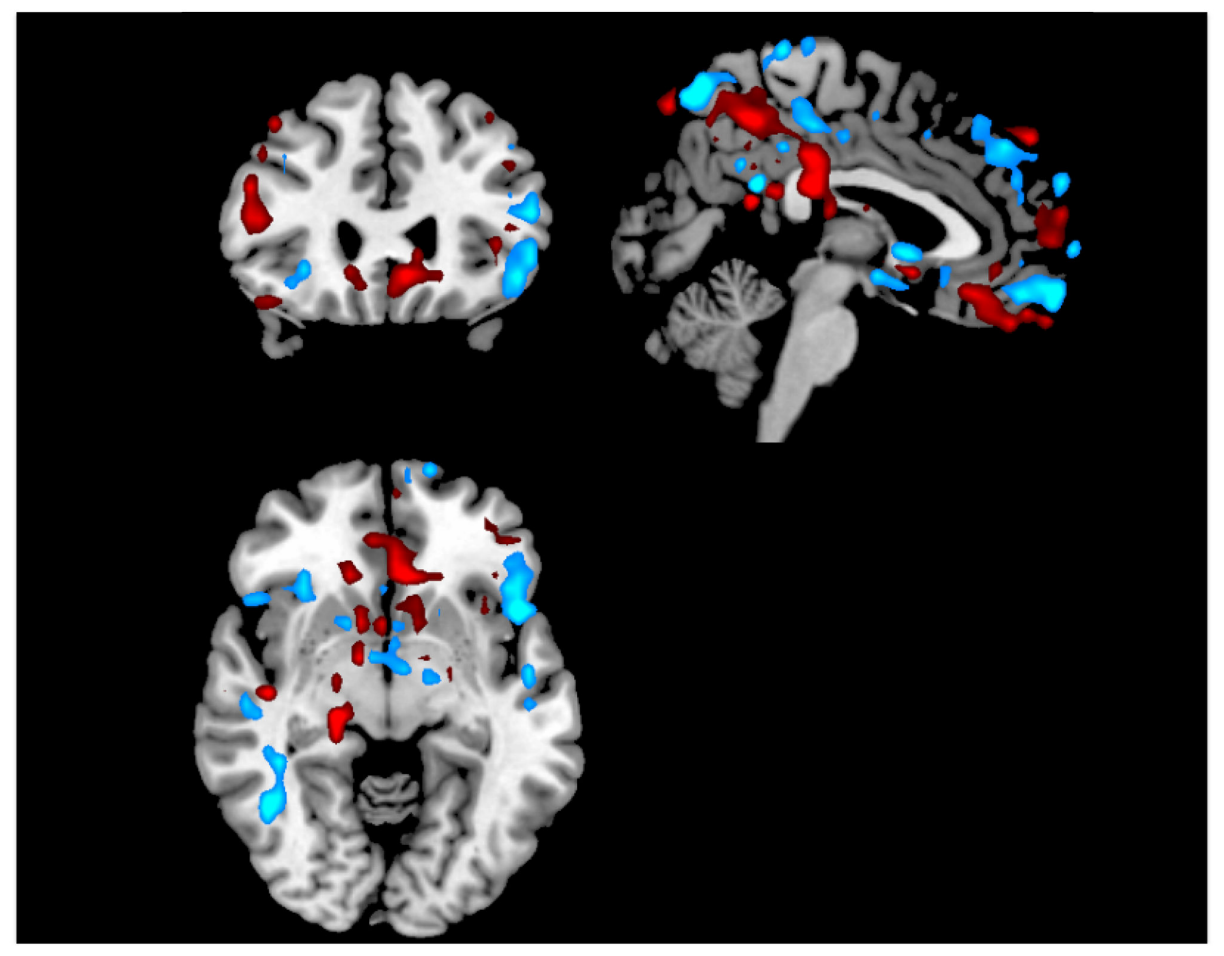
Weight map from the SVM classification of a participant of an emotional memory neurofeedback session. The weights are SVM coefficients determining the discriminant hyperplane, which depicts the relevance of each voxel for the classification between positive and negative conditions. Blue/red colors refer to the sign of these coefficients (negative/positive, respectively). FRIEND saves a NIfTI file containing these maps, which can be viewed using any MRI visualization software. This is only an illustrative map with an arbitrary threshold and slice selection.

SVM classification accuracy in discriminating between the two emotional states tended to increase across three consecutive neurofeedback sessions in three healthy participants (male, 29 y/o: 77%, 85%, 95%; female, 34 y/o: 61%, 62%, 77%; female, 23 y/o: 59%, 70%, 75%). An example of a SVM weight vector map depicting the most discriminant regions in a single participant (male, 29 y/o) is shown in Figure 7. Accurate emotional decoding and modulation have several potential applications, ranging from basic understanding on the role of endogenous modulation of distributed patterns of brain activity associated with specific emotional states, to clinical application in mood disorders [41]. 

As in the previous example, we evaluated the consistency of our real-time approach by comparing SVM projection time series computed with FRIEND’s real-time pipeline with those obtained following offline standard FSL-FEAT preprocessing. The correlations between real-time and offline SVM projections (over time, calculated as described in subsection 2.5.1) were above 0.97 for all three participants.

### ROI-based Functional Connectivity Neurofeedback

In this example, participants (one 43 y/o man, one 30 y/o woman and one 48 y/o woman) identified two types of emotionally salient autobiographical memories associated with either guilt or indignation feelings, and were cued to evoke these memories in the scanner (these comprised the main conditions). As a baseline condition, a mental subtraction task was employed consisting of subtracting 7’s from an initial arbitrary number (e.g. for number 113: 106, 99, 92, etc). Participants were cued visually with keywords (projected on an LCD screen) to engage in the guilt, indignation or subtraction conditions. In the first session (functional localizer), participants engaged in conditions above without neurofeedback. This first run was used to select active voxels (based on the FSL-FEAT routine embedded in FRIEND) within pre-defined anatomical ROIs (MNI coordinates; anterior temporal lobe: x=58, y=0, z=-8, 4mm sphere; subgenual cortex: x=-6, y=26, z=-9, 6mm sphere; coordinates modified from [42]). In the subsequent neurofeedback session, the same design was employed, but this time combined with contingent feedback stimuli (thermometer levels). The thermometer levels reflected the correlation values between ROI time series, updated every TR (see Video S2). 

**Figure 8:**
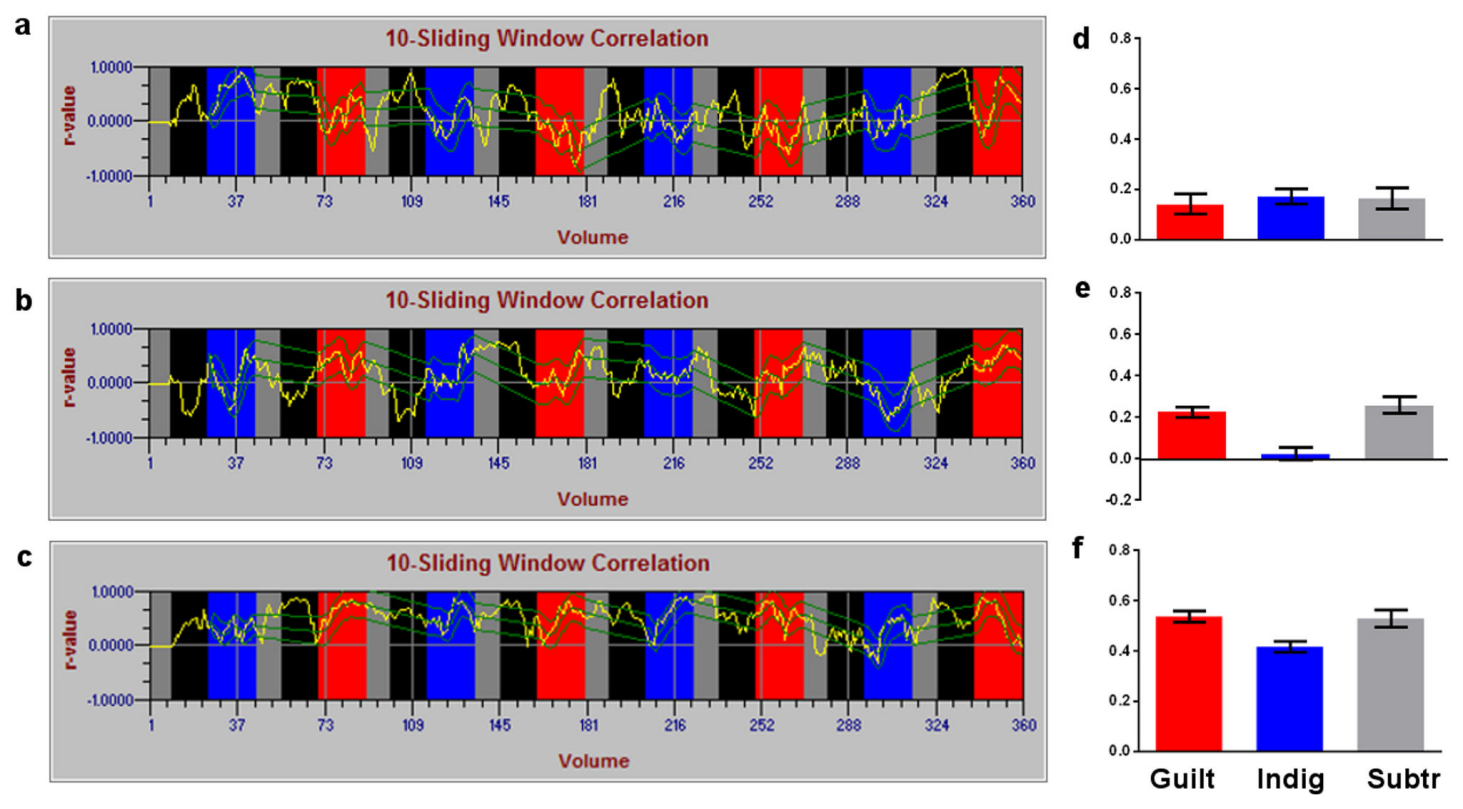
Correlation curves from the second session from all three subjects. Green lines represent head motion (RMS threshold). Gray columns represent subtraction blocks, while emotional blocks are represented in blue (indignation) and red (guilt) columns. The initial five volumes, which are discarded from the correlation calculus, are shown in black. The correlation was computed using a sliding window of 10 volumes.

The functional localizer session comprised two hundred volumes, while the neurofeedback session comprised three hundred and sixty volumes. A block-design was employed, and each emotional block (4 guilt and 4 indignation per run; 15 volumes per block in the first run; 35 volumes per block in the second session, which involved neurofeedback) was interleaved with a subtraction block (8 blocks per session, 10 volumes per block in each session). Real-time ROI-to-ROI correlations from the second session of all three participants are shown (Figure 8). Because head movements can give rise to spurious correlations, a RMS threshold consisting of the average of the previous 40 volumes ± 0.4 absolute RMS value was employed (see Figure 8).

FRIEND real-time implementation was compared to the standard FSL-FEAT offline preprocessing by considering the time-variant values of sliding window correlations from three participants. Pearson correlation coefficients between real-time and offline time-variant correlations (calculated as described in subsection 2.6) were above 0.95 for all participants.

## Discussion

We have introduced FRIEND, a new toolbox enabling real-time fMRI-NFB using single region of interest (based on the level of BOLD activity compared to a baseline signal) or multiple brain regions (using support vector machine-based multivoxel pattern analysis). The GUI runs natively in Microsoft Windows®, with available versions for Apple Macintosh® and Linux platforms. FRIEND is a fully documented and freely available toolbox for the research community, and allows straightforward (1) data preprocessing, (2) feature selection (ROI or GLM-based), (3) SVM training/classification and (4) customized neurofeedback. Furthermore, the close integration of FRIEND with FSL renders it an interesting platform for further developments from a wider community.

FRIEND does not require installation of additional software beyond the operational system, except if users wish to call routines from Matlab for more customized processing. Additionally, FSL and libSVM are embedded in FRIEND, allowing users to have direct access to key parameters of these packages through a simple GUI. Furthermore, for advanced users, full access to advanced configuration parameters is possible through text editing of an ASCII file. FRIEND can employ a technically unlimited number of ROIs for time-course analysis, or SVM-based training and data classification, offering a wide range of options for image visualization, analysis and neurofeedback strategy. The pipeline and most preprocessing steps (e.g., sliding window averaging and spatial smoothing) were designed with the main goal of allowing the handling of noise while keeping flexibility for developing more personalized setups (processing parameters, feedback stimuli, etc). Finally, it is important to highlight some choices made during the development of FRIEND: (i) Delphi/Lazarus language was used because the executable package is fast and allows building flexible graphical user interfaces; (ii) preprocessing steps (motion-correction and anatomical registration) are based on FSL routines, an open source and well-established suite in the neuroimaging community. As herein implemented, it runs in feasible time for real-time applications; (iii) real-time multivoxel pattern classification using SVM is carried out by libSVM, an open, efficient and widely validated library in the academic community.

One key limitation for the development and application of fMRI neurofeedback is the fact that MRI imposes a behaviorally restrictive and non-natural environment and is an expensive technology, in sharp contrast to EEG or near-infrared spectroscopy (NIRS). Nonetheless, fMRI neurofeedback may prove to be useful in establishing anatomically more refined models that could be adapted to more portable technologies such as EEG. In addition, the fast pace of development of immersive, virtual reality technologies will help make the MRI environment a more ecological one. Moreover, there is initial evidence showing that fMRI neurofeedback allows rapid learning, so that participants may “transfer” the acquired ability to modulate their brain states to behavior outside the scanner [23]. 

Functional MRI is perhaps the only technology allowing for measuring brain function non-invasively with reasonable spatial accuracy, including in subcortical regions that are key for cognitive mechanisms such as emotion, motivation, mood and decision-making [43,44]. As such, fMRI neurofeedback is in a unique position to contribute to the understanding of how endogenous, voluntary modulation of brain regions / networks may help improve cognition, emotion and behavior.

Another important aspect is that individual variability of brain states and how they change in response to neurofeedback is a poorly understood issue. This is more critical given the lack of normative databases. This difficulty is due to the early stage of development of this technology, however, and should be lessened by the establishment of standardized protocols and of normative databases [45] for specific brain states that will come along with a wider use of “reverse-inference” type studies and fMRI-neurofeedback. Significant efforts on these aspects are actually under way [44,45]. 

## Conclusion and Future Perspectives

The future use of MRI neurofeedback will critically depend on both technical advances in this technology, as well as on the successes obtained in its experimental and clinical applications [46,47]. Important advances will likely arise from (1) the growing ability to mathematically/topographically represent progressively more complex brain states according to specific cognitive, emotional or motivational domains, (2) the establishment of brain signatures of dysfunctional states associated with neuropsychiatric conditions, as well as of adaptive states that can be enhanced by neurofeedback training and (3) the success of properly designed experimental and clinical, randomized controlled trials in showing the efficacy of this approach. We hope that the availability of new tools, such as the one herein presented, will contribute to a wider use of fMRI neurofeedback in experimental basic and clinical research settings.

## Supporting Information

Video S1 **ROI-based BOLD level feedback.** The video shows a motor task experiment where input parameters (reference anatomical and functional images, TR, number of functional volumes, baseline condition, etc) are entered in the FRIEND interface before clicking the “Feedback” button on the control window. The condition blocks are specified in the ASCII file at the “Design” field. Motion parameters are shown in the first three graphs (from top to bottom) during real-time acquisition and processing. ROI-based BOLD signal and percent signal change are shown on the bottom graph. Task instructions and neurofeedback figures (shown to participants via projection) can also be observed by the experimenter by choosing the NFB Figures option in the Display menu. In this particular example, a thermometer is used to provide the neurofeedback. The thermometer describes the ratio [(average BOLD signal of the ROI) – (average BOLD signal of the ROI during the previous baseline condition)] / (average BOLD signal of the ROI during the previous baseline condition). BOLD signal (red line) and % signal change (green) are shown in bottom left panel. Average % signal change is provided at the end of the session (right panel).
(MP4)

Video S2 **Correlation-based feedback.** The video shows an emotion task experiment (autobiographical memories associated with either guilt or indignation). Input parameters (reference anatomical and functional images, TR, number of functional volumes, baseline condition, etc) are entered in the FRIEND interface before clicking the Feedback button on the control window. Motion parameters are shown in the first three graphs during real-time acquisition and processing (rotation and translation for x, y and z in blue, green and red, respectively; the first volume of the run is assumed as the reference image). The average image signal across voxels contained in each of the two ROIs is shown in the fourth graph (re-scaled using Z-transformation). The bottom graph shows a sliding window correlation between the two ROIs in yellow. The green line represents a moving average of the real-time correlation (± one standard deviation), defining an interval for maximum and minimum values of the feedback scale (thermometer). The experimenter can visualize the task instruction and neurofeedback figures by clicking on the “NFB Figures” option in the Display menu. In this particular example, a thermometer is used to provide neurofeedback to the participant. During excessive motion, the thermometer is frozen and turns into gray to warn participants.
(MP4)

Video S3 **SVM-based neurofeedback session.** Input parameters (reference anatomical and functional images, TR, number of functional volumes, baseline condition, etc) need to be entered into the FRIEND interface before clicking the “Feedback” button on the control window. Motion parameters are shown in the first three graphs during real-time acquisition and processing. This video also shows the “Display Brain Slices” functionality, which allows dynamic visualization of signal changes overlaid on a participant’s coregistered anatomical image. The arbitrary signal threshold can be modified using the “Inf” (inferior level) and “Sup” (superior level) buttons in the control box. These buttons define the value range of the color bar. In this particular example, neurofeedback is provided to the participant using the ring figures, whose color is associated with the condition (positive or negative emotion), level of distortion is dependent on the multivariate distance of the data from the SVM discriminating hyperplane (projection values, i.e., the decision value given by its position relative to the classification boundary), which is used to inform participants about their own performance.
(MP4)
